# Single-Component Elastic Biocarbon Aerogel with Reversibly Mechanotunable Electrical and Thermal Conductivities for Dual-Mode Pressure–Temperature Sensing

**DOI:** 10.1021/acsami.5c23402

**Published:** 2026-01-21

**Authors:** Xiang Li, Shaoqi He, Yintong Huang, Gaoqiang Xu, Yankun Zhou, Chengxuan Tang, Xiqiang Zhong, Xiaoyu Zhao, Hirotaka Koga

**Affiliations:** † Zhejiang Key Laboratory of Energy Conversion Materials for Advanced Motor, College of Materials and Environmental Engineering, 12626 Hangzhou Dianzi University, No. 115 Wenyi Road, Xihu Zone, Hangzhou 310012, China; ‡ 162755 The Third Affiliated Hospital of Wenzhou Medical University, Wenzhou 325200, China; § SANKEN (The Institute of Scientific and Industrial Research), The University of Osaka, 8-1 Mihogaoka, Ibaraki, Osaka 567-0047, Japan

**Keywords:** dual-mode pressure−temperature sensing, elastic biocarbon aerogel, reversibly mechanotunable conductivities, ordered pore structures, chitin nanofibers

## Abstract

Flexible dual-mode pressure–temperature sensors enable the construction of soft and smart miniature electronics and have been fabricated using assemblies with intricate structures and multiple active components derived from limited resources but are challenging to realize using a single active component derived from sustainable resources. Herein, a crab shell-derived chitin nanofiber dispersion is subjected to directional freeze-drying followed by morphology-retaining pyrolysis to afford a single-component elastic biocarbon aerogel with a high compressibility, robust elasticity, and reversibly mechanotunable pore structure and electrical and thermal conductivities. Owing to its low through-plane thermal conductivity (0.031 W m^–1^ K^–1^) in the pristine (uncompressed) state and pressure-dependent electrical conductivity, this aerogel enables temperature-invariant dynamic pressure sensing with a sensitivity of up to 36.8 kPa^–1^ at a unilateral heating temperature of up to 100 °C. Upon switching between compressed (80% strain) and uncompressed (0% strain) states, the temperature sensitivity of the aerogel alternates between 0.44 and 0.01 °C^–1^, respectively, because of the concomitant reversible change in thermal conductivity (0.031 and 0.223 W m^–1^ K^–1^, respectively). The developed aerogel provides a simple, robust, and sustainable platform for dual-mode pressure–temperature sensing in on-skin health monitoring, smart tactile electronics in soft robotics, etc.

## Graphical abstract

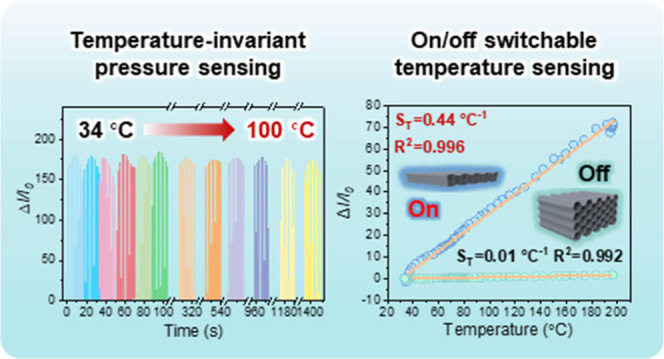

## Introduction

Flexible electronics is rapidly evolving toward greater intelligence and miniaturization, with wearable sensors playing pivotal roles in on-skin health monitoring,^1^ human–machine interactions,^2^ and smart robotics.^3^ Given that unilateral temperature and dynamic pressure are critical parameters for physiological state and environmental interaction assessment, their dual-mode sensing is necessary for precise medical diagnostics and intelligent perception systems.^4,5^ Temperature sensors rely on the thermally induced changes in the concentration or mobility of carriers in the active material, often requiring active materials with high thermal expansion coefficients or thermoelectric effects,^6^ while pressure sensors rely on the modulation of electron conduction pathways through mechanoinduced interfaces or changes in the internal contact area.^7^ However, the realization of concomitant temperature and pressure sensing is hindered by mutual interference due to temperature fluctuations inducing resistance signal drift in pressure sensors and mechanical deformation disturbing the readings of temperature sensors.^8^ Therefore, most dual-mode pressure–temperature sensors require complex discrete assemblies to separately output signals.^9^ The development of dual-mode devices based on single-component materials and capable of switchable pressure and temperature sensing is highly challenging but desirable for the integration and miniaturization of wearable sensors.

The development of dual-mode temperature–pressure sensors largely relies on the design of active material composition and structure.^2^ Regarding composition design, piezoresistive (temperature-insensitive) and thermoelectric (pressure-insensitive) composites are often used as active materials to sense pressure and temperature, respectively.^10^ That is, the active materials are engineered to facilitate either charge carrier transport (piezoresistive component) or phonon-mediated heat transport (thermoelectric component) in response to stimuli, which enables selective output signal modulation. Yin et al.^11^ fabricated a polyurethane/carbon nanofiber sponge coated with graphene, achieving a pressure-sensing performance with a negligible temperature interference. In this case, the metal-like electrical conductivity of graphene helped suppress electrical resistance changes in response to thermal stimuli. Thus, compositing metal-like electrical components in active materials can minimize their thermally induced electrical resistance changes. The structural design of active materials is an emerging strategy for converting volume resistance into surface resistance and thus weakening the effects of pressure on temperature sensing.^12^ Hu et al.^13^ created a dual-mode Ti_3_C_2_T_*x*_/poly­(3,4-ethylenedioxythiophene)/poly­(styrenesulfonate) (PEDOT/PSS) sensor that featured top and bottom layers with mulberry- and rose-like microstructures, respectively, and thus separated temperature- and pressure-sensing signals. The bottom layer exhibited marked changes in contact area and electrical resistance under pressure, while the top layer exhibited a rapid change in the thermoelectric response to temperature. This structural design enabled the dual-mode sensing of pressure and temperature. However, the above-mentioned strategies rely on integrating thermoelectric and piezoresistive active components, multisignal output, and discrete sensor design,^14^ which hinders device miniaturization and compromises reliability. Thus, single-component structure-engineered devices capable of dual-mode pressure–temperature sensing are required to realize smart and miniature flexible electronics.

Carbon-based porous materials with tunable pore structures and electrical conductivities have emerged as an ideal platform for fabricating multifunctional sensors.^15^ Porous carbons (e.g., graphene,^16^ carbon nanotubes,^17^ and carbon nanofibers^18^) can be composited with elastic matrices (e.g., polydimethylsiloxane,^19^ polyurethane,^20^ and polyimide^21^) to improve the sensitivity and mechanical durability of pressure sensors and can exploit nanoscale phonon scattering effects or the Seebeck effect of thermoelectric materials (e.g., PEDOT/PSS) to achieve high temperature sensitivities.^22^ Shen’s group fabricated a dual-mode pressure–temperature sensor by coating porous melamine foam with single-walled carbon nanotubes and PEDOT/PSS, with the low thermal conductivity and excellent compressibility of the active material-coated melamine foam yielding a low temperature detection limit (0.03 K) and rapid pressure response (120 ms).^23^ Thus, carbon-based materials with purposefully designed three-dimensional porous structures exhibit low thermal conductivities, which can be used to increase the temperature difference for temperature sensing and enhance compressibility for pressure sensing. However, most conventional carbon-based pressure–temperature sensors comprise multiple components, are obtained from fossil-derived carbon precursors, and require multiple signal output channels, with the thermoelectric and piezoresistive components outputting voltage and current signals, respectively.^15^ In addition, temperature-induced resistance changes remain unseparated for pressure sensing.^12^ From the perspectives of sensor miniaturization, sustainability, and simplification, the rational pore structure tuning of biomass-derived carbon (biocarbon) is a desirable strategy for fabricating single-component dual-mode pressure–temperature sensors with a single signal output channel.

Herein, the above-mentioned strategy was adopted to fabricate single-component biocarbon aerogels with reversibly mechanotunable pore structures via the high-temperature pyrolysis of aerogels with ordered pore structures produced through the directional freeze-drying of aqueous bionanofiber (chitin, cellulose, or silk nanofiber) dispersions. Owing to the high thermal stability of chitin nanofibers, the pyrolysis of the corresponding aerogel proceeded with morphology retention. The corresponding biocarbon aerogel exhibited a high compressibility and elasticity and achieved dual-mode (switchable) sensing of pressure and temperature through the mechanotuning of its pore structure upon compression and release. The thermally insulating nature and mechanically tunable electrical conductivity of this aerogel enabled temperature-invariant dynamic pressure sensing at unilateral temperatures of up to 100 °C. The developed aerogel enabled direct on/off switchable temperature sensing based on compression strain control. Specifically, the aerogel was sensitive to temperature stimuli at 80% strain but insensitive at 0% strain, as pore structure compression enhanced heat conduction. Both pressure- and temperature-sensing signals were output by a sole current change, which simplified sensor design. These features render the developed biocarbon aerogel a robust and sustainable single-component platform for advanced applications, such as on-skin wearable sensors and smart soft robotics.

## Results and Discussion

### Morphology-Retaining Pyrolysis of the Bionanofiber Aerogels

The biocarbon aerogels were prepared according to the method described in our previous report.^24^ First, aqueous bionanofiber (chitin, cellulose, or silk nanofiber) dispersions were fabricated into aerogels via freeze-casting and freeze-drying (Figure 1). The resulting bionanofiber aerogels (Figure S1) showed similar microscale pore structures, featuring pore sizes of several tens of microns and pore walls formed by bionanofiber networks (Figure 2). The Fourier transform infrared (FT-IR) spectrum of the cellulose nanofiber aerogel showed typical cellulose peaks. The spectrum of the chitin nanofiber aerogel showed amide I (1660 and 1620 cm^–1^) and II (1560 cm^–1^) peaks characteristic of α-chitin.^25^ The spectrum of the silk nanofiber aerogel featured a broad peak centered at 1511 cm^–1^ and attributed to β-sheet structures (Figure S2).^26^

**Figure 1:**
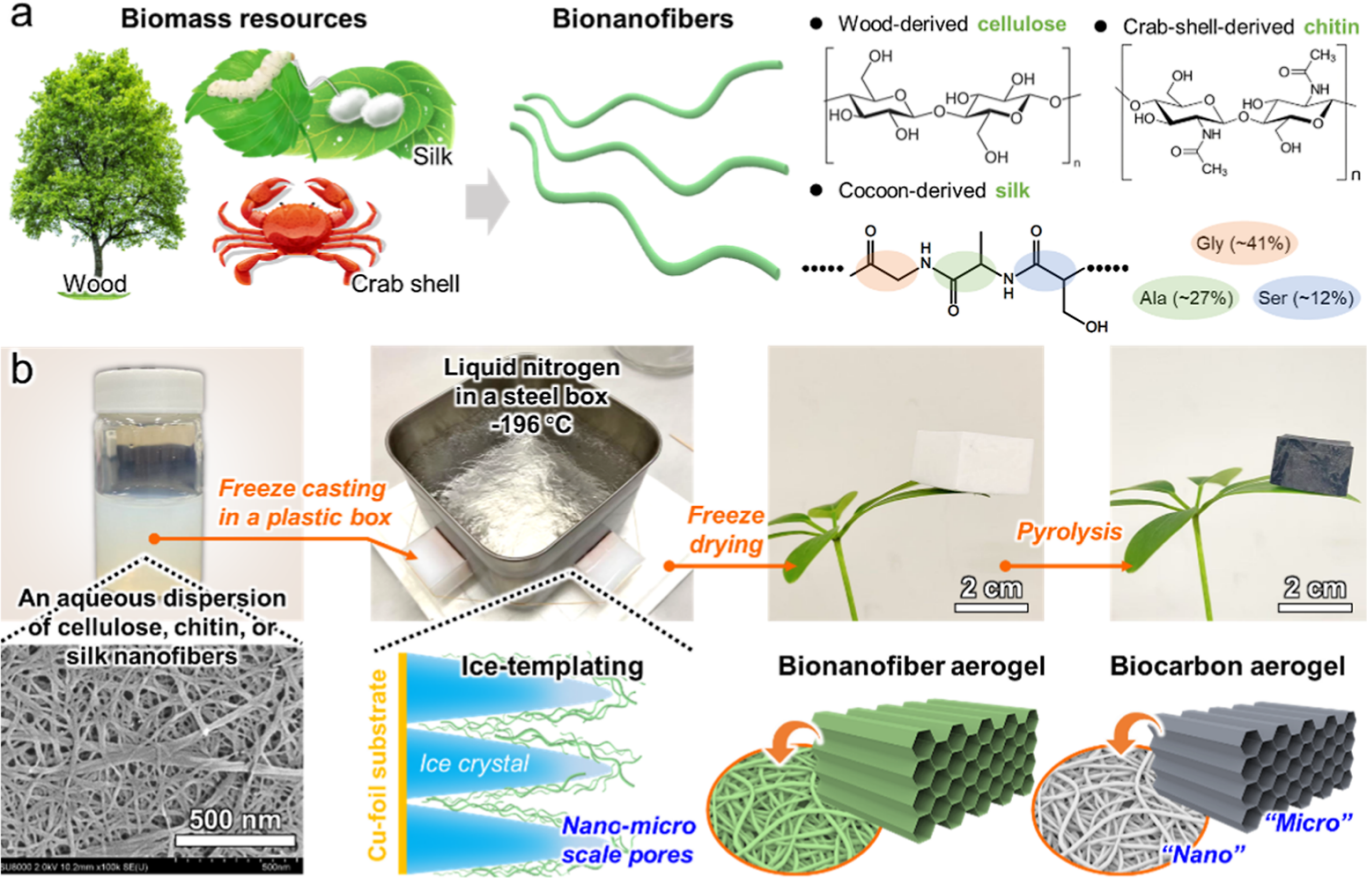
Schematics of (a) biomass resources; cellulose, chitin, and silk nanofibers; and nanofiber molecular structures and (b) biocarbon aerogel preparation via the Cu foil-based directional freeze-drying of aqueous bionanofiber dispersions and subsequent pyrolysis.

**Figure 2:**
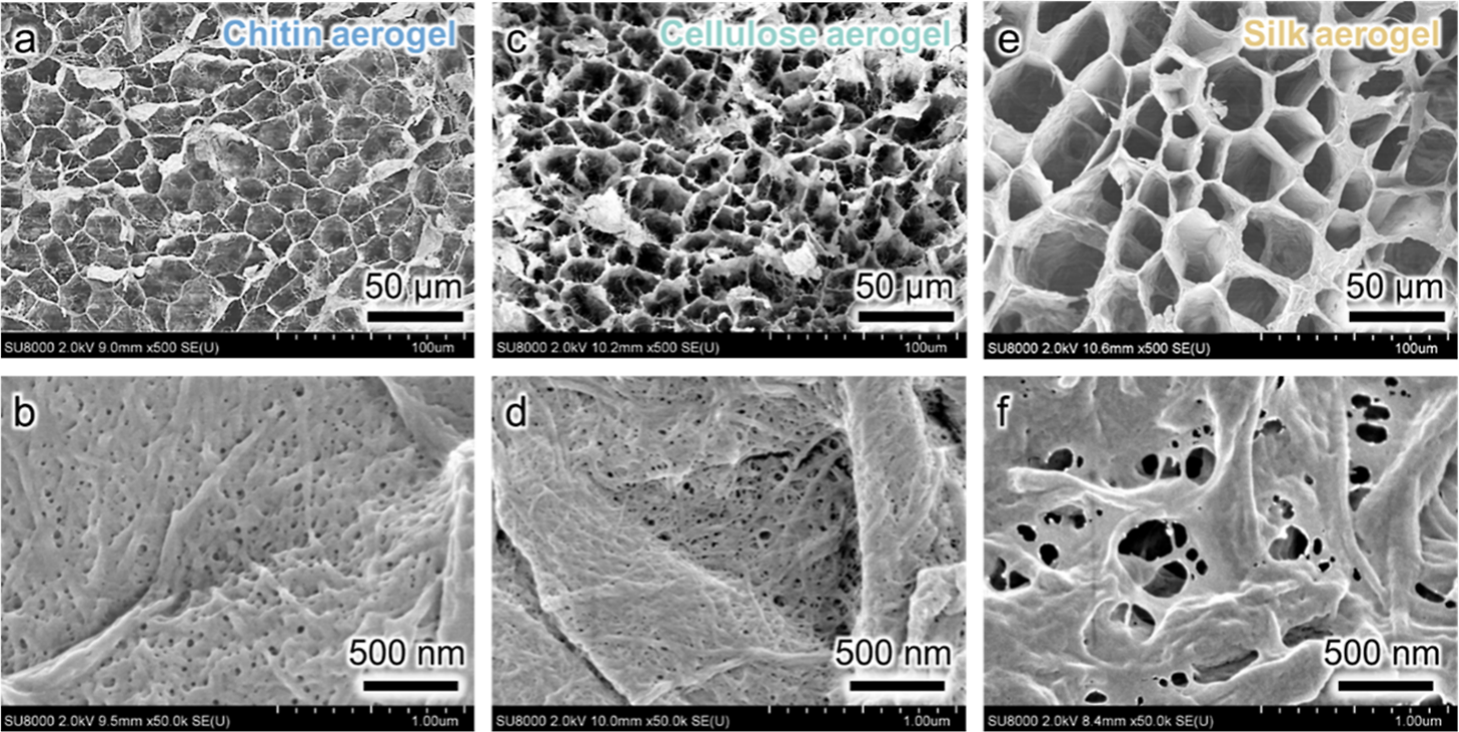
Field-emission scanning electron microscopy (FE-SEM) images presenting the (a,c,e) microscale and (b,d,f) nanoscale pore structures derived from nanofiber networks in the microscale pore walls of (a,b) chitin, (c,d) cellulose, and (e,f) silk nanofiber aerogels.

Subsequently, the bionanofiber aerogels were subjected to high-temperature pyrolysis to prepare the biocarbon aerogels (Figure 1). Unlike the chitin nanofiber-derived biocarbon aerogel, which had a regular cubic shape (Figure 3a), the cellulose nanofiber-derived biocarbon aerogel showed substantial cracking and volume shrinkage (Figure 3b). The silk nanofiber-derived biocarbon aerogel showed an even greater volume shrinkage and did not retain the cubic shape of the precursor aerogel (Figure 3c). The biocarbon aerogels retained the nanofiber network morphology (Figure 3d–f), although the nanofiber width decreased after pyrolysis (see also Figure 2b,d,f). The chitin nanofiber-derived biocarbon aerogel retained the original microscale pore structure after pyrolysis (Figures 2b and 3d), thus showing a better morphology retention than its cellulose and silk nanofiber counterparts.

**Figure 3:**
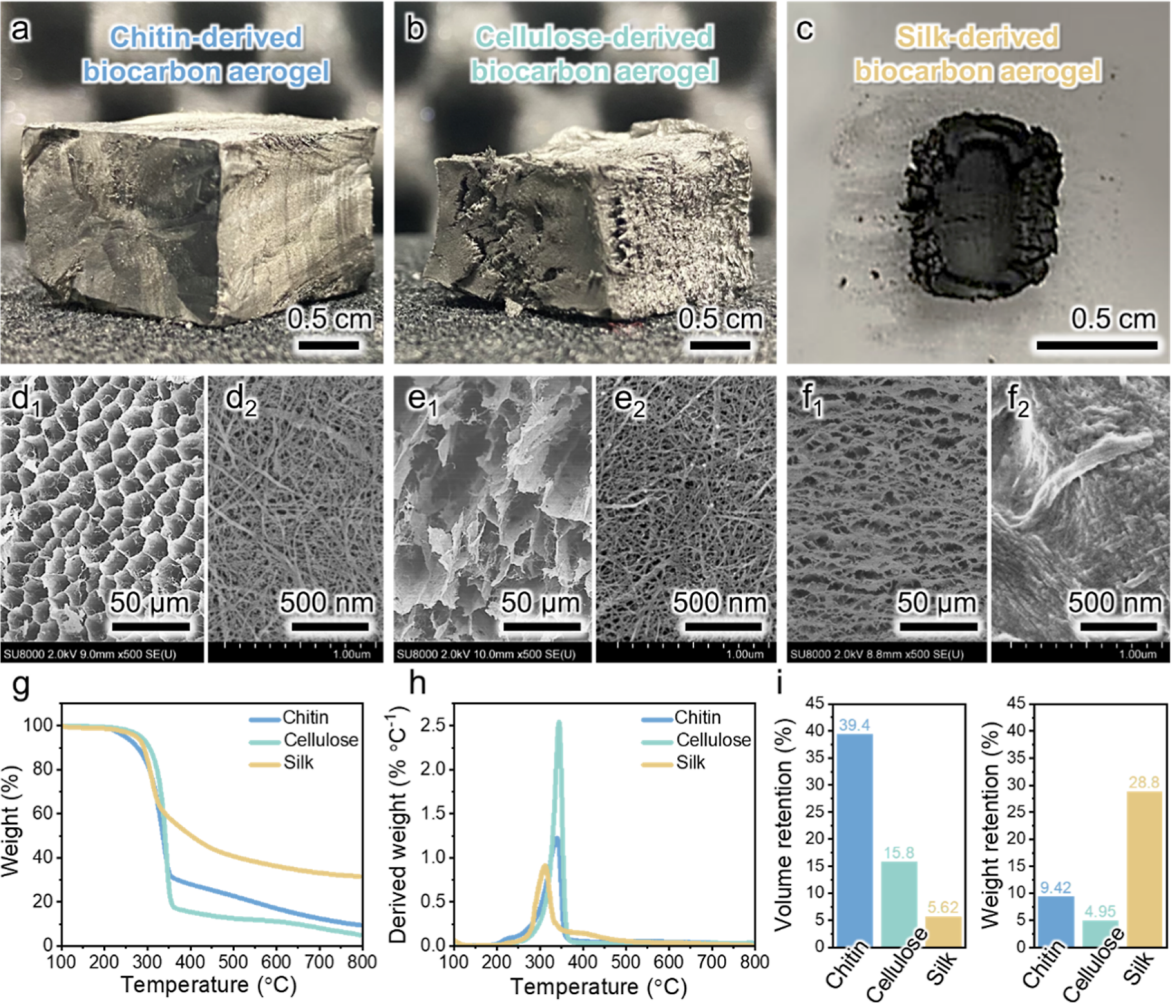
Photographs of biocarbon aerogels derived from (a) chitin, (b) cellulose, and (c) silk nanofibers. FE-SEM images presenting the (d_1_–f_1_) microscale and (d_2_–f_2_) nanoscale pore structures derived from nanofiber networks in the microscale pore walls of the biocarbon aerogels derived from (d_1_,d_2_) chitin, (e_1_,e_2_) cellulose, and (f_1_,f_2_) silk nanofibers. (g) Thermograms, (h) differential thermogravimetry curves, and (i) volume and weight retentions of the bionanofiber aerogels.

The three bionanofibers exhibited a degradation onset temperature of ∼200 °C (Figure 3g). The cellulose and chitin nanofibers showed similar derivative thermogravimetry (DTG) peaks at ∼345 °C (Figure 3h). However, the chitin nanofibers showed higher weight (9.42%) and volume (39.4%) retentions than the cellulose nanofibers (4.95% and 15.8%, respectively) after pyrolysis at 800 °C (Figure 3i), which indicated that volatile substance generation and resulting shrinkage during the thermal decomposition of the former were less pronounced than those observed for the latter.^27^ These results indicate that the chitin nanofibers had a higher thermal stability than the cellulose nanofibers,^28^ possibly due to the acetyl-amino group of chitin.^29^ The silk nanofibers showed a DTG peak at ∼311 °C, thus featuring the lowest thermal stability. Even though the silk nanofibers had the highest weight retention of 28.8%, their low volume retention of 5.62% substantially hindered morphology preservation upon pyrolysis. The exceptional weight retention of the silk nanofibers was ascribed to their high heteroatom content, with N doping stabilizing the carbon backbone and N-containing volatile formation increasing the yield of carbon during pyrolysis.^30^ The low volume retention of the silk nanofibers was ascribed to their largely amorphous structure, with the facile decomposition of the amorphous regions during pyrolysis causing polypeptide chain contraction and densification.^31^ Therefore, the chitin nanofibers exhibited the best morphology retention performance because of their highest thermal stability. The FT-IR spectra of the biocarbon aerogels indicated that pyrolysis resulted in the carbonization of the bionanofibers and weakening of hydrogen bonds between them^32^ (Figure S3). Hence, the chitin nanofiber-derived biocarbon aerogel was chosen for further investigations.

### Pore Structure Tailoring of the Biocarbon Aerogels

The morphology retention ability of chitin nanofibers enabled the pore structure design of the corresponding biocarbon aerogel. Three freeze-casting methods, namely ordinary freezing in liquid N_2_ (Figure 4a) and directional freezing outside liquid N_2_ without (Figure 4b) and with (Figure 4c) Cu foil, were applied to the aqueous chitin nanofiber dispersion to modulate ice crystal nucleation and growth. For ordinary freezing in liquid N_2_ (−196 °C), the ice crystals initially formed in all directions of the aqueous chitin nanofiber dispersion and rapidly grew inward until the dispersion was fully frozen. Directional freezing outside liquid N_2_ provided a unidirectional temperature gradient, promoting ice crystal growth from low to high temperatures.^33^

**Figure 4:**
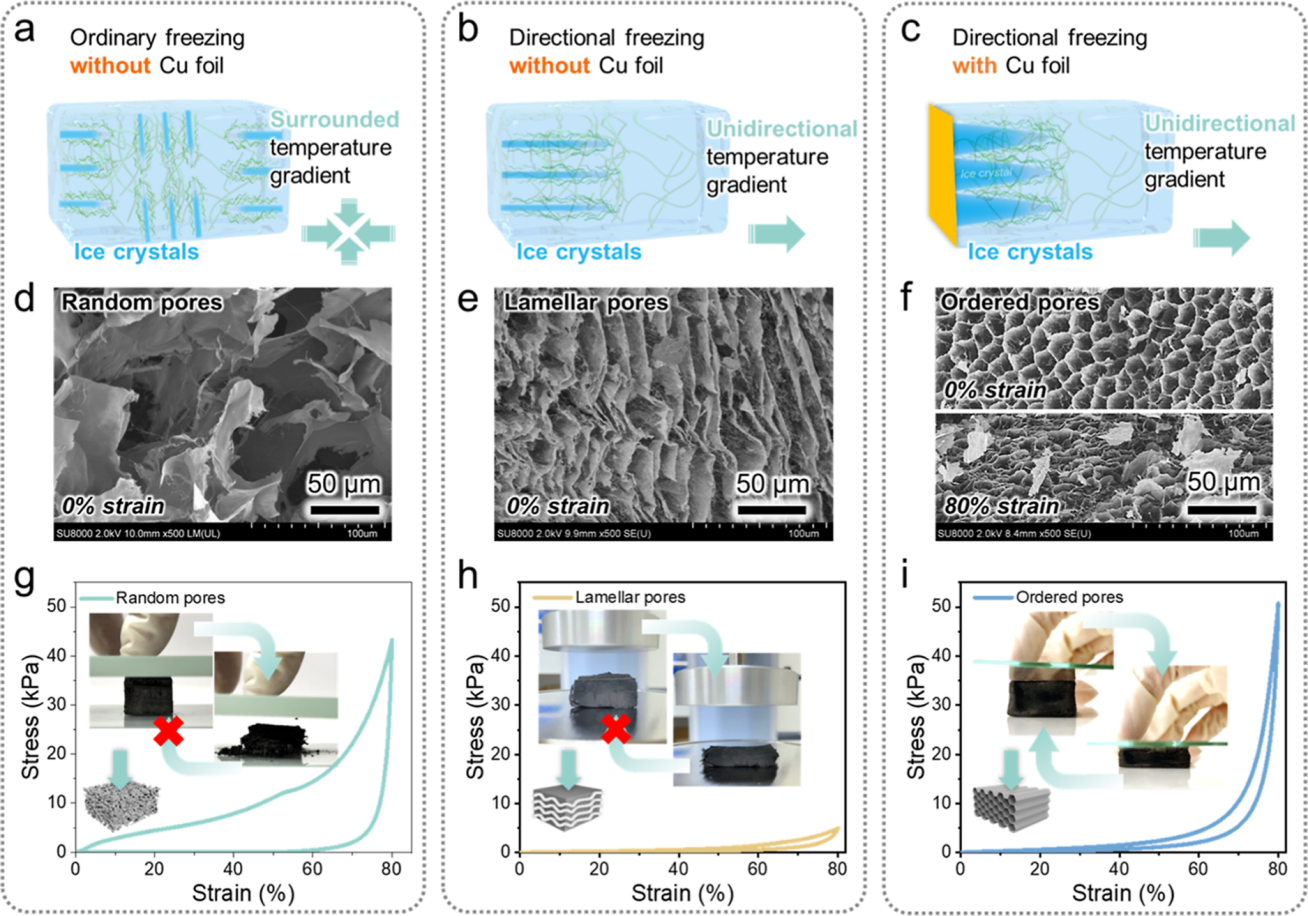
Mechanical properties of chitin nanofiber-derived biocarbon aerogels with different pore structures. (a–c) Schematics and (d–f) FE-SEM images of biocarbon aerogels prepared by (a,d) ordinary freezing and directional freezing (b,e) without and (c,f) with Cu foil at compression strains of (d–f) 0 and (f) 80%. Stress–strain curves of the biocarbon aerogels with (g) random, (h) lamellar, and (i) ordered pores recorded during one compression loading (to 80% strain)–unloading cycle.

Ordinary freezing generated random pores (Figure 4d), whereas directional freezing afforded regular lamellar (Figure 4e) or ordered (Figure 4f) pores. These results suggest that directional freezing helped regulate the ordered growth of ice crystals in one direction. Figure 4b,e suggest that the ice crystals nucleated and grew in an in-plane fashion, and the water-insoluble chitin nanofibers were confined between the in-plane ice crystals to form lamellar structures.^34^ However, in the presence of Cu foil, out-of-plane ice crystals nucleate and grow vertically on it (Figure 4c). These out-of-plane ice crystals compel the chitin nanofibers to form continuous fibrous networks that act as the walls of the honeycomb channels (Figure 1b). After freeze-drying and high-temperature pyrolysis, a biocarbon aerogel with uniform, honeycomb-like, and anisotropic ordered microscale pores can be obtained (Figure 4f, and see Figure S4 for more details). Thus, the morphology retention ability of the chitin nanofibers helped tailor the pore structures of the corresponding biocarbon aerogel.

To demonstrate the importance of pore structure tailoring, we examined the mechanical properties of biocarbon aerogels with different pore structures. Figure 4g–i shows the stress–strain curves of biocarbon aerogels with random, lamellar, and ordered pore structures recorded during compression to 80% strain followed by recovery. For the aerogels with lamellar and ordered pore structures, compression was performed in the direction perpendicular to these structures. The biocarbon aerogel with random pores exhibited a maximum stress of ∼40 kPa, collapsing upon compression to 80% strain (Figure 4g). The biocarbon aerogel with lamellar pores sustained a stress of <5 kPa and exhibited poor recovery to its original shape, thus showing a low mechanical resistance against compression (Figure 4h). On the contrary, the biocarbon aerogel with ordered pores sustained a high stress of up to 50 kPa and exhibited a small hysteresis area and good recovery to its original shape, showing superior elastic properties with an excellent compressibility (Figure 4i). These results indicate that the honeycomb-like ordered pore structures efficiently dissipated compression stress concentration and guided reversible deformation upon compression to 80% strain (Figure 4f), as reported for a cellulose aerogel with an anisotropic hierarchical pore architecture.^35^ Thus, the morphology retention ability of the chitin nanofibers and formation of ordered pore structures enabled the realization of an elastic biocarbon aerogel.

### Elastic Properties and Fatigue Resistance of the Biocarbon Aerogel with Ordered Pore Structures

The biocarbon aerogel with ordered pore structures could be compressed without brittle fracture and completely recovered its original shape (Figure 5a). High-speed camera images showed that this aerogel (∼0.13 g) could rebound a metal component (∼6.8 g, Figures 5b and S5) with a fast recovery rate (∼1600 mm s^–1^) (Video S1), outperforming state-of-the-art fossil-derived carbon aerogels with random,^36^ cell-like,^37^ lamellar,^38^ centripetal,^39^ cross-linked,^17^ foam-like,^40^ and polar bear hair-like^41^ pore structures (Figure 5c and Table S1). Thus, the chitin nanofiber-derived biocarbon aerogel exhibited a superior elasticity and spring-like instantaneous recovery.

**Figure 5:**
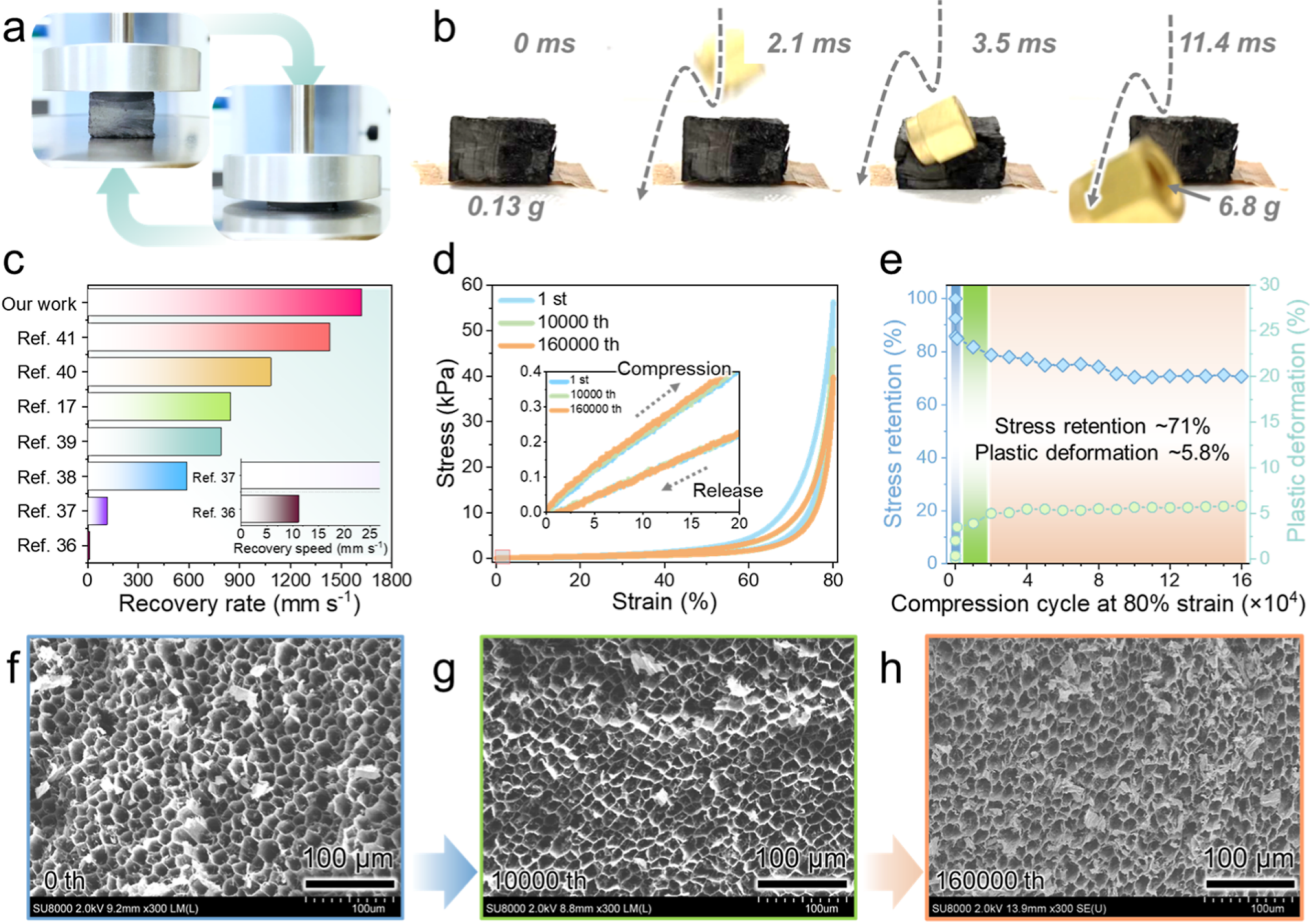
High-speed camera images of the chitin nanofiber-derived biocarbon aerogel captured during compression and release in response to pressure applied by (a) a compression test fixture and (b) a rebounding metal object. (c) Recovery rates of previously reported aerogels and the chitin nanofiber-derived biocarbon aerogel. (d) Stress–strain curves and (e) stress retention and plastic deformation of the biocarbon aerogel recorded during 160,000 compression (80% strain)-release cycles. FE-SEM images showing the ordered pore structures of the biocarbon aerogel (f) before cycling and after (g) 10,000 and (h) 160,000 compression-release cycles.

The crescent-shaped stress–strain curve of the biocarbon aerogel (Figure 5d) was distinct from those of conventional open-cell foams,^42^ showing three characteristic regions, namely (i) an initial linear elastic region related to the minimal bending of the thin pore walls,^38^ (ii) a flat plateau region related to the buckling of the thin pore walls or yielding of the discontinuous nanofiber networks with plastic deformation,^43^ and (iii) a sharp increase region related to the densification of the honeycomb-like ordered pore structures (Figures 4f and S6). Furthermore, the biocarbon aerogel displayed narrow hysteresis loops upon cyclic compression during fatigue testing (160,000 compression (80% strain)-release cycles), thus showing robust elasticity due to reversibly mechanotunable ordered pore structures.

Although the biocarbon aerogel showed almost overlapping hysteresis loops, it experienced stress reduction (∼20%) and plastic deformation (>2%) during 10 compression (80% strain)-release cycles (Figure 5e) because of the presence of internal stress. The stress reduction and plastic deformation became more pronounced upon further cycling (up to 10,000 cycles), possibly because of the structural damage of the honeycomb-like ordered pore structures (Figure 5f,g). Between 10,000 and 160,000 cycles, the stress reduction and plastic deformation slowed down and subsequently reached saturation (Figure 5e). The biocarbon aerogel maintained its microscale pore structures even after 160,000 cycles despite experiencing further structural damage (Figure 5h). These results indicate that the robust elasticity of the biocarbon aerogel was provided by its soft and thin pore walls derived from the weak interactions between biocarbon nanofibers, as well as its honeycomb-like ordered pore structures (Figures S3 and S4). Thus, the reversibly mechanotunable pore structures of the biocarbon aerogel provided an excellent elasticity and fatigue resistance over 160,000 compression-release cycles.

### Temperature-Invariant Pressure-Sensing Performance of the Biocarbon Aerogel

The biocarbon aerogel exhibited reversible electrical conductivity changes upon compression and release (0.33 S m^–1^ at 0% strain, 3.26 S m^–1^ at 80% strain), showing piezoresistive behavior (Figure S7a,b). The current and temperature changes of the biocarbon aerogel were recorded simultaneously at an applied direct-current voltage of 1 V during compression-release cycling upon heating by a hot plate coupled with a digital temperature meter (Figure 6a). Figure 6b shows the real-time current changes at a constant temperature of 25 °C during compression from 0% to 80% strain and subsequent release, with the static strain (i.e., pressure) maintained for 1 min. The pressure applied to the biocarbon aerogel was matched with that observed in the stress–strain curves (Figure 5d). The current remained constant under static pressure and sharply increased with an increase in pressure, which indicated that the biocarbon aerogel could respond to both static and dynamic pressure changes. Figure 6c shows the results of pressure sensitivity calculations, revealing that the biocarbon aerogel was highly sensitive to small pressure increases and decreases (*S* of up to 36.8 kPa^–1^). Notably, the pressure sensitivity in the pressure decrease stage exceeded that in the pressure increase stage. This result was ascribed to the changes in the plane-to-plane, plane-to-point, and point-to-point contact areas within the biocarbon aerogel in the pressure decrease stage exceeding those in the pressure increase stage, thereby resulting in a larger Δ*I*/*I*_0_. This phenomenon can be further understood by considering the stress–strain curves of the biocarbon aerogel (Figure 5d), in which case the mechanical energy stored during the compression (pressure increase) stage facilitated the recovery of the deformed pore structures in the release (pressure decrease) stage. To improve the applicability and durability of the biocarbon aerogel-based sensor, an additional long-term pressure sensing durability test was conducted with cyclic compression-release at 80% strain. The biocarbon aerogel-based sensor retained a stable signal amplitude and baseline with negligible drift after 160,000 cycles of compression-release at 80% strain, demonstrating its excellent durability and operational repeatability (Figure S8).

**Figure 6:**
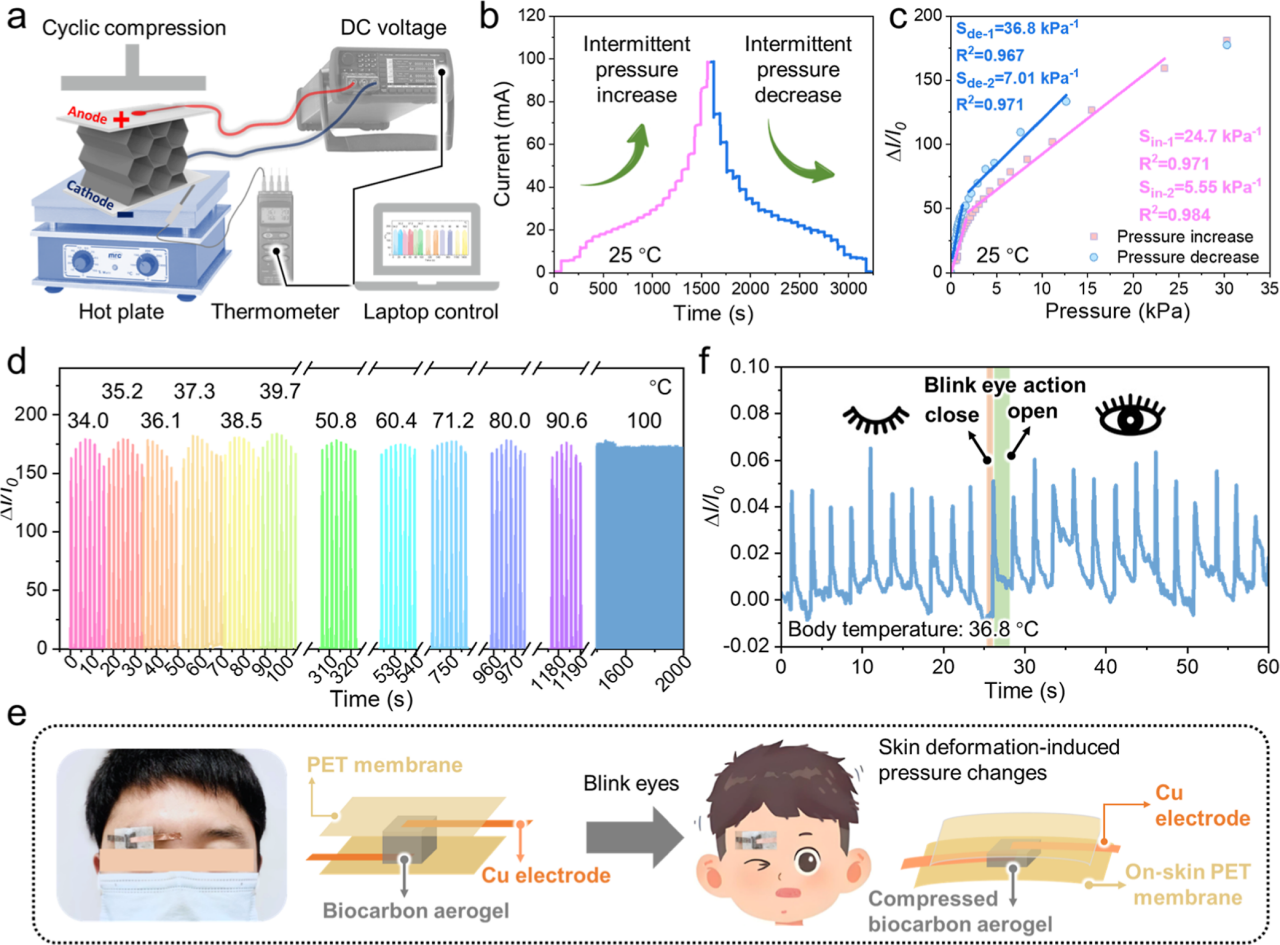
(a) Schematic of the experimental setup used to test the dynamic pressure-sensing performance of the unilateral heated chitin nanofiber-derived biocarbon aerogel with ordered pore structures. (b) Current changes at 25 °C upon intermittent pressure increase and decrease, with each pressure stage lasting 1 min. (c) Relative current change (Δ*I*/*I*_0_) and pressure sensitivity (*S*_p_) at 25 °C in the pressure range of 0–30 kPa. (d) Δ*I*/*I*_0_ at unilateral heating temperatures from 34 to 100 °C during compression (80% strain)-release cycling. (e) Schematic structure of the on-skin wearable pressure sensor and mechanism of sensing pressure upon blinking. (f) Δ*I*/*I*_0_ during blinking (body surface temperature = 36.8 °C).

Temperature is a key factor interfering with the accuracy of pressure sensing. In our previous work, a nanochitin-derived carbon aerogel was obtained by tailoring chitin nanofiber concentration and carbonization temperatures, which exhibited pressure sensitivity in an ultrawide temperature range of −196 to 600 °C because of its antifreezing and flame-retardant properties.^32^ However, for practical applications, such as real-time health monitoring and soft robotics, the pressure-sensing performance of the biocarbon aerogel should be systematically investigated upon a continuous temperature change. Furthermore, in numerous scenarios (e.g., on-skin health monitoring and tactile electronics in soft robotics), one side of the sensor is exposed to heat.^44^ Therefore, pressure sensors that can withstand unilateral thermal disturbance are required. Figure 6d shows the real-time Δ*I*/*I*_0_ values of the biocarbon aerogel during compression (80% strain)-release cycling upon unilateral heating at 34.2–100 °C. In this figure, each peak corresponds to one compression-release cycle.^45,46^ The Δ*I*/*I*_0_ values substantially increased during compression and rapidly decreased during release, indicating a rapid current response. The current response and Δ*I*/*I*_0_ values remained almost constant during unilateral heating, even when unilateral heating at 100 °C was maintained for >600 s. This result indicates the temperature-invariant pressure-sensing performance of the biocarbon aerogel. The corresponding mechanism is thought to dominated by the mechanotunable electrical conductivity (Figure S7) and low original through-plane thermal conductivity (0.031 W m^–1^ K^–1^ at 0% strain, Figure S9) of the aerogel. Owing to the abundant air within the biocarbon aerogel, heat was difficult to store during compression-release cycling even upon unilateral heating at 100 °C. The thermally-induced electrical conductivity variation is negligible compared to the pressure-induced one at unilateral heating temperatures of up to 100 °C, ensuring that the pressure signal is predominantly governed by mechanical effects. Therefore, the biocarbon aerogel-based pressure sensor was deemed to be suitable for measuring pressure at unilateral temperatures ranging from 34.2 to 100 °C without the need for additional compensation techniques.

Considering its temperature-invariant pressure-sensing ability and high pressure sensitivity, the biocarbon aerogel was used as an on-skin wearable pressure sensor to detect human physiological signals, such as blinking and vocal cord vibrations. Figure 6e shows the pressure sensor structure used to detect blinking. The sensor was attached to the eyebrow area in the eyes-open state and experienced compression upon eye closure because of the skin deformation-induced pressure change. The related Δ*I*/*I*_0_-time plot exhibited regular peaks, each corresponding to a single blink, as reported previously (Figure 6f).^47^ The baseline and amplitude of the Δ*I*/*I*_0_ peaks for each blinking action were similar, indicating sensor stability.^48^ Vocal cord vibrations were identified by attaching the sensor to the throat, which indicates the suitability of the developed aerogel for voice recognition devices (Figure S10). Thus, the biocarbon aerogel was demonstrated to be suitable for use in on-skin wearable pressure sensors and soft pressure sensors capable of withstanding unilateral thermal disturbance.

### On/Off Switchable Temperature-Sensing Performance of the Biocarbon Aerogel

As mentioned above, the biocarbon aerogel enabled temperature-invariant dynamic pressure sensing despite unilateral-temperature changes because of its low original through-plane thermal conductivity (0.031 W m^–1^ K^–1^ at a compression strain of 0%). Indeed, the uncompressed biocarbon aerogel provided an excellent through-plane thermal insulation, with its top-surface temperature saturating at ∼33 °C upon unilateral heating at 200 °C from the bottom surface regardless of the glass plate substrate (Figures 7a and S11). Based on the side-view temperature gradient image of the uncompressed biocarbon aerogel (Figure 7b), its top-layer temperature was estimated as ∼50 °C, confirming the excellent thermal insulation ability. The through-plane thermal insulation properties of the uncompressed biocarbon aerogel originated from its highly porous structures and low density of 7.4 mg cm^–3^ (Figure S12). The abundant air within the aerogel induced radiation-mediated thermal conduction, thereby leading to a high thermal insulation performance^49^ (Figure S13). On the contrary, the compressed biocarbon aerogel (80% strain) showed a higher top-layer temperature (144.8 °C, Figure 7c) and through-plane thermal conductivity (0.223 W m^–1^ K^–1^, Figure S9). Upon compression to 80% strain, most of the air was expelled, which facilitated thermal conduction (Figure S13). Thus, these results indicate the mechanotunable thermal conductivity of the biocarbon aerogel.

**Figure 7:**
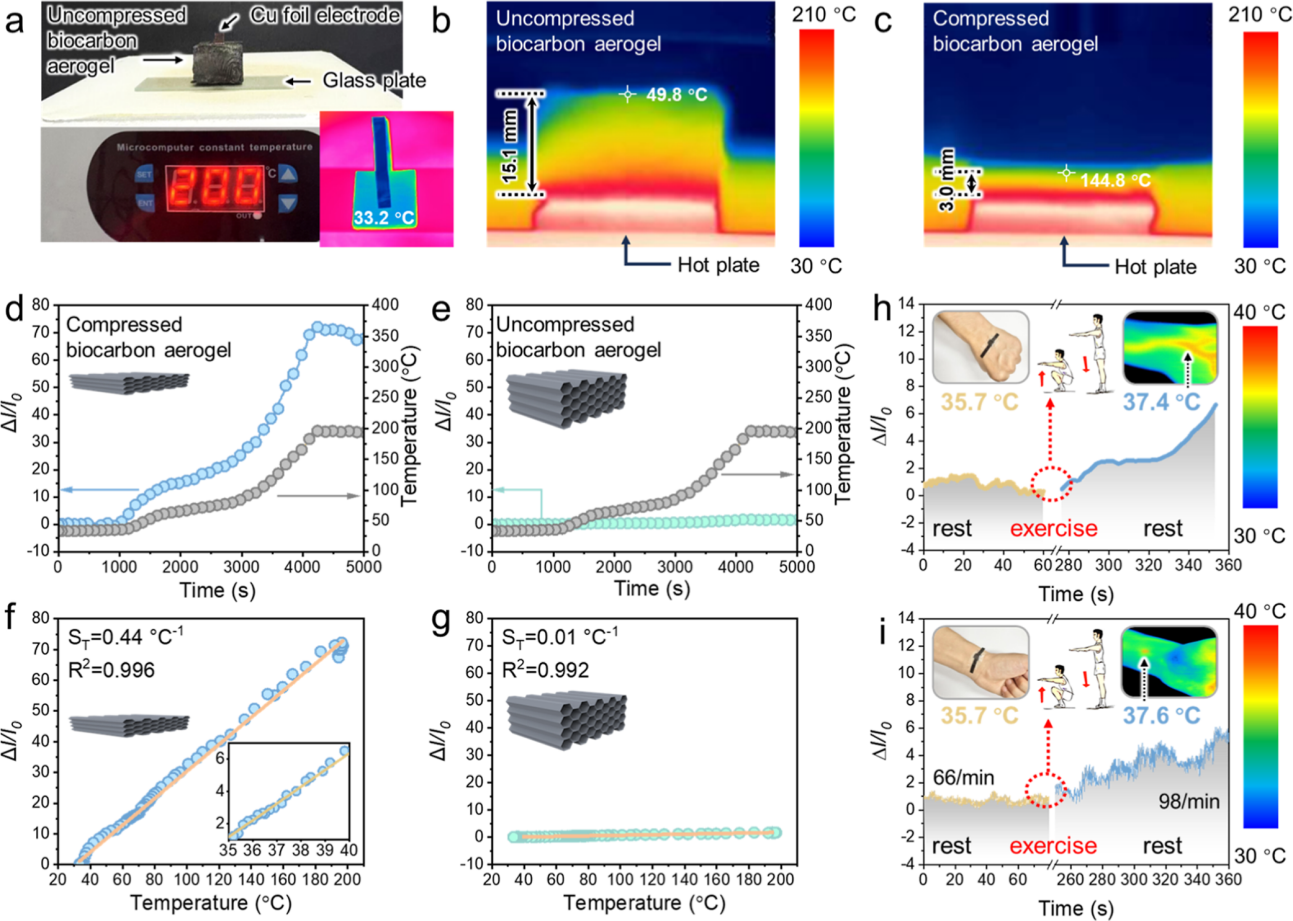
(a) Optical image of the biocarbon aerogel-based temperature sensor in the uncompressed state upon unilateral heating at 200 °C and top-view IR false-color images of the sensor after unilateral heating at 200 °C for 5 min (saturated top-surface temperature = 33.2 °C). Side-view IR false-color images of (b) uncompressed (0% strain) and (c) compressed (80% strain) biocarbon aerogels after unilateral heating at 200 °C for 5 min. Changes in the Δ*I*/*I*_0_ and unilateral temperature of (d) compressed and (e) uncompressed biocarbon aerogels. Δ*I*/*I*_0_ versus unilateral temperature plot and temperature sensitivity (*S*_T_) of (f) compressed and (g) uncompressed biocarbon aerogels. Δ*I*/*I*_0_ of the compressed (80% strain) biocarbon aerogel-based temperature-pressure sensor attached to the (h) back of the hand and (i) palm side of the wrist during squat exercises and rest periods (inset: temperature of hand skin after sensor detachment measured by an IR camera).

To demonstrate the importance of this mechanotunable thermal conductivity, we compared the temperature-sensing performances of the compressed and uncompressed biocarbon aerogels. Figure 7d and e show the Δ*I*/*I*_0_–time plots obtained for the compressed and uncompressed biocarbon aerogels, respectively, unilaterally heated from 30 to 200 °C. The compressed biocarbon aerogel showed a gradual increase in Δ*I*/*I*_0_ to 72.2 with an increase in the unilateral heating temperature to 200 °C (Figure 7d). The Δ*I*/*I*_0_–time curve resembled the temperature–time curve, indicating the temperature-dependent nature of the electrical conductivity^50^ of the compressed aerogel. This temperature sensitivity was ascribed to the enhanced through-plane carrier transport within the aerogel (Figure 7c).^7^ By contrast, the Δ*I*/*I*_0_ of the uncompressed biocarbon aerogel was almost constant (0–0.16) despite an increase in unilateral temperature to 200 °C (Figure 7e), as this aerogel showed through-plane thermal insulation properties (Figure 7a,b). The Δ*I*/*I*_0_ of the compressed and uncompressed aerogels showed a good linear relationship with unilateral temperature (Figure 7f,g), with the temperature sensitivity of the former (0.44 °C^–1^) markedly exceeding that of the latter (0.01 °C^–1^). Thus, the simple mechanical compression and release of the biocarbon aerogel enabled on/off switchable temperature sensing, a feature that can benefit intermittent temperature monitoring in on-skin wearable electronics and smart soft robotics.

Finally, the compressed (80% strain) biocarbon aerogel was used for on-skin temperature–pressure sensing to determine the human body temperature and pulse rate. The compressed biocarbon aerogel–based sensor was attached to the back of a human hand to detect skin temperature before and after a squat exercise (inset of Figure 7h). A relatively stable Δ*I*/*I*_0_ of 1–2 was observed before the exercise, increasing to 3–6 after the exercise. According to the Δ*I*/*I*_0_–temperature correlation of the sensor (inset of Figure 7f), the corresponding skin temperatures were determined as 35–36 and 37–40 °C, respectively. According to the IR images, the skin temperature after sensor detachment was 35.7 and 37.4 °C before and after the exercise, respectively, in line with the increase detected by the sensor. The skin temperature determined by the sensor after the exercise exceeded that extracted from the corresponding IR images, possibly because the sensor attachment trapped heat. These results indicate that the sensor could detect changes in skin temperature. To simultaneously determine the body temperature and pulse rate before and after the squat exercise, the sensor was attached to the palm side of the wrist (Figure 7i). As in the case when the sensor was attached to the back of the hand, the baseline of the Δ*I*/*I*_0_–time profile gradually increased after the exercise, indicating successful temperature sensing. Furthermore, the sensor displayed regular Δ*I*/*I*_0_ variations over time, with each variation corresponding to a single pulse signal. Prior to the exercise, the pulse rate equaled 66 min^–1^, which falls within the normal heart rate range for adults.^51^ After the exercise, the pulse rate increased to 98 min^–1^, which indicated successful pulse rate sensing before and after exercise. Thus, the sensor could simultaneously detect skin temperature and pulse signals, which demonstrates the applicability of the compressed biocarbon aerogel for the real-time on-skin monitoring of temperature and pressure-related physiological signals. The sensor exhibited good repeatability across multiple samples fabricated under identical conditions; there was minimal variation in key performance metrics such as the current response baseline and Δ*I*/*I*_0_ values. This consistency underscores the reliability of the fabrication method. Additionally, the sensor demonstrated stable sensing performance after repeated attachment and detachment cycles, confirming its operational stability for wearable applications.

The developed biocarbon aerogel-based sensor offered pressure and temperature sensitivities of up to 36.8 kPa^–1^ and 0.44 °C^–1^, respectively. Furthermore, the sensor required only a single signal output channel; both pressure- and temperature-sensing signals were output by a sole current change. These features were positively comparable to those of the representative dual-mode pressure–temperature sensors (Table 1).^22,23^ The biocarbon aerogel can be fabricated from renewable biomass resources, providing high-performance, simple, and sustainable sensors.

**Table 1:** Comparison of the Developed Biocarbon Aerogel-Based Sensor to the Representative Dual-Mode Pressure–Temperature Sensors

material	pressure sensitivity (kPa^–1^)	temperature sensitivity (°C^–1^)	signal output channel number	refs
chitin nanofiber–derived biocarbon aerogel	36.8	0.44	1	this work
PEDOT/PSS/nanofibrillated cellulose/glycidoxypropyl trimethoxysilane aerogel	0.87^a^	∼0.4^a^	2	^22^
PEDOT/PSS/single-walled carbon nanotube/melamine foam	10.8	∼0.2^a^	2	^23^

a These sensitivity values were estimated from the data given in the corresponding references.

## Conclusion

A single-component biocarbon aerogel capable of switchable pressure and temperature sensing was fabricated via the directional freeze-drying of aqueous chitin nanofiber dispersions followed by pyrolysis. The chitin nanofibers enabled morphology retention during pyrolysis and, hence, the tailoring of the pore structures in the resulting biocarbon aerogel. The biocarbon aerogel with tailored honeycomb-like ordered pore structures exhibited a high compressibility, robust elasticity, and reversibly mechanotunable electrical (0.33 and 3.26 S m^–1^ at 0% and 80% strain, respectively) and thermal (0.031 and 0.223 W m^–1^ K^–1^ at 0% and 80% strain, respectively) conductivities. Owing to its mechanotunable electrical conductivity and low original thermal conductivity, the biocarbon aerogel offered a temperature-invariable sensitivity of up to 36.8 kPa^–1^ for dynamic pressure detection at unilateral heating temperatures of 30–100 °C. The aerogel also exhibited temperature sensitivities of 0.01 and 0.44 °C^–1^ at compression strains of 0 and 80%, respectively, because of its mechanotunable thermal conductivity, thus enabling on/off switchable temperature sensing. Furthermore, the aerogel was successfully used in on-skin wearable sensors capable of simultaneously detecting pressure and temperature. Further challenges remain in achieving fully decoupled, accurate, and self-powered sensing of pressure and temperature. These features render the chitin nanofiber-derived biocarbon aerogel a simple, smart, and sustainable platform for advanced applications, such as on-skin wearable electronics and soft robotics.

## Experimental Section

### Biocarbon Aerogel Fabrication

Biocarbon aerogels were prepared from aqueous bionanofiber dispersions via freeze-casting, freeze-drying, and high-temperature pyrolysis as described elsewhere (Figure 1).^24^ Crab shell-derived chitin nanofibers (BiNFi-s chitin, SFo-20002, 2.0 wt %), wood-derived cellulose (BiNFi-s cellulose, WFo-10002, 2.0 wt %), and cocoon-derived silk nanofibers (BiNFi-s silk, KCo-30005, 5.6 wt %) were supplied by Sugino Machine, Ltd. (Namerikawa, Japan) and dispersed in water at a concentration of 1.0 wt %. The dispersions were vacuum-centrifuged at 1400 rpm and 25 °C for 5 min to remove air bubbles using a defoaming apparatus (ARV-930TWIN, Thinky Corp., Tokyo, Japan). Each defoamed dispersion was poured into a handmade mold (length × width × depth: 30 mm × 30 mm × 20 mm) made of a 1.0 mm-thick acrylic plate (AcrySunday Co., Ltd., Osaka, Japan). An adhesive Cu foil (No. 8701-00, Maxell Sliontec, Ltd., Kanagawa, Japan) was preattached to the inner wall (length × depth: 30 mm × 20 mm) of the mold to facilitate directional freezing. The Cu foil side of the filled mold was brought into contact with the outside surface of a liquid N_2_-containing open steel box, and directional freezing was conducted for 30 min. The fully frozen sample was freeze-dried (Scient-10N, Ningbo Scientz Biotechnology Co., Ltd., Ningbo, China) for 60 h below −60 °C and 6.2 Pa to obtain the cellulose, chitin, or silk nanofiber aerogel. Each aerogel was pyrolyzed in a tube furnace (OTF-1200X, HF-Kejing Materials Technology Co., Ltd., Hefei, China) under N_2_ by heating to 500 °C at 2 °C min^–1^, holding for 2 h, heating from 500 to 800 °C at 5 °C min^–1^, holding for 1 h, and cooling to room temperature at 2 °C min^–1^.

The chitin nanofiber dispersion was also subjected to random and directional freezing without the Cu foil. Random freezing was performed by immersing the dispersion-filled mold into liquid N_2_. Directional freezing without the Cu foil was performed by attaching the dispersion-filled mold to the outside wall of a liquid N_2_-filled steel box. The resulting chitin nanofiber aerogels were pyrolyzed. The dispersion concentration and freeze-drying and pyrolysis conditions were identical to those used for directional freezing with the Cu foil.

### Evaluation of the Elastic Properties and Fatigue Resistance of the Biocarbon Aerogels

The elastic properties of the biocarbon aerogels were evaluated using an Instron universal testing system equipped with a 500 N load cell (Instron 3367, Instron Co., Norwood, USA). The aerogels were cut into cuboids (15 mm × 15 mm × 10 mm) and placed between two compression stages, with the upper stage used to apply and release uniaxial compression. The compression-release axis was perpendicular to the anisotropic microscale pore channel direction of the aerogel. The stress–strain hysteresis curves were recorded at a maximum strain of 80% and stroke speed of 10 mm min^–1^ under ambient conditions (25 °C). The compressive fatigue resistance was evaluated by subjecting the aerogels to 160,000 compression (80%)-release cycles at a frequency of 0.4 Hz using a modified desktop endurance testing motor. The stress and height retentions (*R*) were quantified as the value measured at cycle *n* (*X*_*n*_) divided by the corresponding initial value (*X*_0_)­

1 $$R=\frac{X_{n}}{X_{0}}$$

### Evaluation of the Temperature-Invariant Pressure-Sensing Performance of the Biocarbon Aerogels

A temperature-invariant pressure sensor was fabricated by cutting the biocarbon aerogel into a cuboid (15 mm × 15 mm × 10 mm) and placing it between two Cu foil electrodes attached to a 1 mm-thick glass plate (Shangying Quartz Products Co., Ltd., Jinzhou, China). The sensor bottom was fixed on an electronically controlled hot plate (ND-1A, AS ONE Co., Osaka, Japan) and unilaterally heated by the same at a rate of 2 °C min^–1^. The hot plate surface temperature was monitored using a thermometer (MT-309, MotherTool Co., Ltd., Nagano, Japan). The pressure-sensing performance of the biocarbon aerogel at different temperatures was examined using the above-mentioned universal testing system and an electrochemical workstation (ModuLab XM ECS, Solartron Analytical-AMETEK Advanced Measurement Technology Inc., Berkshire, UK) under an applied direct-current voltage of 1 V. Pressure was applied in the direction perpendicular to the tailored anisotropic microscale pore channel direction of the aerogel while its compression strain was increased from 0% to 80%.

The relative change in current (Δ*I*/*I*_0_) was calculated as

2 $$\Delta I/I_{0}=\frac{I-I_{0}}{I_{0}}$$

where *I*_0_ and *I* are the currents in the absence and presence of applied pressure, respectively. The pressure sensitivity (*S*_P_, kPa^–1^) was calculated as

3 $$S_{P}=\frac{\delta \left(\Delta I/I_{0}\right)}{\delta P}$$

where δ­(Δ*I*/*I*_0_) is the change in Δ*I*/*I*_0_ in response to a change in the applied pressure (δ*P*, kPa). In other words, *S*_P_ corresponds to the slope of the Δ*I*/*I*_0_ vs *P* plot.

An on-skin wearable pressure sensor was fabricated by sandwiching the biocarbon aerogel (5 mm × 5 mm × 5 mm) between two adhesive Cu foil electrodes and sealing with two flexible poly­(ethylene terephthalate) membranes (Zhonglian Electronic Materials Co., Ltd., Dongguan, China). The method for on-skin blinking and vocal cord vibrations pressure sensing was approved by the Hangzhou Dianzi University Ethics Committee and complied with the research regulations of Hangzhou Dianzi University at 2025. Informed consent from the no-skin sensor participant was also obtained prior to the experiment.

### Evaluation of the Temperature-Sensing Performance of the Biocarbon Aerogels

The biocarbon aerogel-based temperature sensor was assembled similarly to the corresponding pressure sensor. The uncompressed (0% strain) or compressed (80% strain) aerogel was sandwiched between two Cu foil electrodes attached to a glass plate and fixed on an electronic hot plate. The bottom-electrode side of the aerogel was unilaterally heated from 30 to 200 °C by the hot plate at a rate of 2 °C min^–1^. The hot plate surface temperature was monitored using a thermometer. The temperature-sensing performance was evaluated using the above-mentioned electrochemical workstation at an applied voltage of 1 V. Δ*I*/*I*_0_ was calculated as in eq 2, where *I*_0_ and *I* are the currents at 30 °C and a certain applied temperature, respectively.

The temperature sensitivity (*S*_T_, °C^–1^) was calculated as

4 $$S_{T}=\frac{\delta \left(\Delta I/I_{0}\right)}{\delta T}$$

where δ*T* (°C) is the change in the applied temperature. In other words, *S* corresponds to the slope of the Δ*I*/*I*_0_ vs *T* plot.

An on-skin temperature–pressure sensor was fabricated by sandwiching the compressed (80% strain) biocarbon aerogel between two adhesive Cu foil electrodes and sealing with two poly­(ethylene terephthalate) membranes. The method for on-skin pressure–temperature sensing was approved by the Hangzhou Dianzi University Ethics Committee and complied with the research regulations of Hangzhou Dianzi University at 2025. Informed consent from the no-skin sensor participant was also obtained prior to the experiment.

### Characterization

Aerogel morphologies were characterized by FE-SEM (SU-8000, Hitachi High-Tech Science Co., Tokyo, Japan) at an accelerating voltage of 2 kV. Prior to FE-SEM imaging, the samples were sputter-coated with Pt at 20 mA for 10 s using an ion sputterer (E-1045, Hitachi High-Tech Science Co., Tokyo, Japan). The pore size distributions of the biocarbon aerogels were estimated from their FE-SEM images using the ImageJ software (USA). HR-TEM imaging was performed at 200 kV (JEM-ARM200F, JEOL, Ltd., Tokyo, Japan) to observe the nanopores and nanofibrous networks of the biocarbon aerogels. FT-IR spectra were recorded using a Nicolet iS instrument (Thermo Fisher Scientific Inc., Waltham, USA) with a scan range from 400 to 4000 cm^–1^. Thermogravimetric analysis was carried out by heating to 800 °C under Ar (100 mL min^–1^) with a heating rate of 10 °C min^–1^ (SDT650, TA Instruments, New Castle, DE, USA). The aerogel volume retention was estimated based on the change in volume due to pyrolysis. The surface temperatures of the uncompressed and compressed aerogels were monitored by an IR camera (VarioCAM, InfraTec, Dresden, Germany). The through-plane thermal conductivity of the aerogels in the direction perpendicular to the anisotropic pore channel direction was measured at 60 °C and compression strains of 0% and 80% using an instrument equipped with a flatbed module (TPS 2500S, Hot Disk Instrument, Gothenburg, Sweden).

## Supplementary Material





## Notes

Figures S1–S13: original shape of bionanofiber aerogels; Fourier transform infrared (FT-IR)/attenuated total reflection spectra of chitin, cellulose, and silk nanofiber aerogels; FT-IR/attenuated total reflection spectra of chitin, cellulose, and silk nanofiber-derived biocarbon aerogels; field-emission scanning electron microscopy (FE-SEM) images of microscale pores, high-resolution transmission electron microscopy images of the nanoscale pores, and size distributions of the biocarbon aerogel; weight of the chitin nanofiber-derived biocarbon aerogel; FE-SEM images of the chitin nanofiber-derived biocarbon aerogel in the direction parallel to the ordered pores; relative current change (Δ*I*/*I*_0_) of the biocarbon aerogel during 160,000 cycles of compression-release at 25 °C and 80% strain. Electrical conductivities and putative mechanism of the mechanotunable electrical conduction of the chitin nanofiber-derived biocarbon aerogel; through-plane thermal conductivities of the chitin nanofiber-derived biocarbon aerogel; real-time current changes recorded by the biocarbon-based pressure sensor upon the periodical pronunciations; IR false-color image of the chitin nanofiber-derived biocarbon aerogel; volumes, weights, and densities of the chitin nanofiber-based aerogel and corresponding biocarbon aerogel; putative mechanism of the mechanotunable thermal conduction of the biocarbon aerogel upon compression and release. Recovery speeds of the elastic chitin nanofiber-derived biocarbon aerogel and state-of-the-art elastic carbon aerogels (PDF)
Video S1. Demonstration of the superelastic of chitin nanofiber-derived biocarbon aerogel (MP4)
