# Supplementary material for: Single-Component Elastic Biocarbon Aerogel with Reversibly Mechanotunable Electrical and Thermal Conductivities for Dual-Mode Pressure–Temperature Sensing
Source: ACS Appl Mater Interfaces. 2026 Jan 21;18(4):7294–306. doi: 10.1021/acsami.5c23402 (PMC12884450; doi:10.1021/acsami.5c23402)
Supplement: Supplementary file 1 [file am5c23402_si_001.pdf]

Supporting Information

Single-Component Elastic Biocarbon Aerogel with Reversibly Mechanotunable Electrical and Thermal Conductivities for Dual-Mode Pressure–Temperature Sensing

Xiang Li [a§], Shaoqi He [b§], Yintong Huang [c], Gaoqiang Xu [a], Yankun Zhou [a], Chengxuan Tang [b], Xiqiang Zhong [b], Xiaoyu Zhao [a*], and Hirotaka Koga [c*]*

[a] Zhejiang Key Laboratory of Energy Conversion Materials for Advanced Motor, College of Materials and Environmental Engineering, Hangzhou Dianzi University, No. 115 Wenyi Road, Xihu Zone, Hangzhou, 310012, China

[b] The Third Affiliated Hospital of Wenzhou Medical University, Wenzhou 325200, China

[c] SANKEN (The Institute of Scientific and Industrial Research), The University of Osaka, 8-1 Mihogaoka, Ibaraki, Osaka 567-0047, Japan

[§] X. L. and S. H. contributed equally to this work.

** Corresponding Authors*

Xiang Li

** E-mail address: lixiang3271@hdu.edu.cn*

Xiaoyu Zhao

** E-mail address: zhaoxy@hdu.edu.cn*

Hirotaka Koga

** E-mail address: hkoga@eco.sanken.osaka-u.ac.jp*

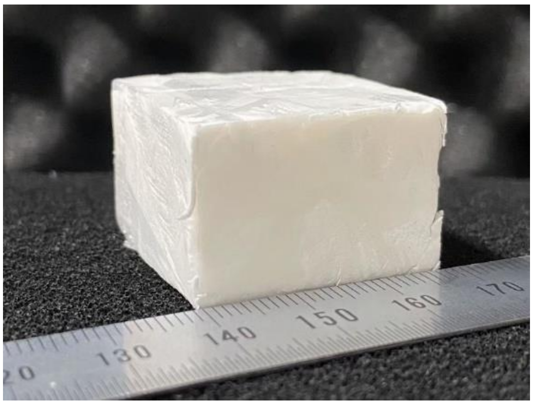

Figure S1. Original shape of bionanofiber aerogels (length = 30 mm, width = 30 mm, height = 20 mm).

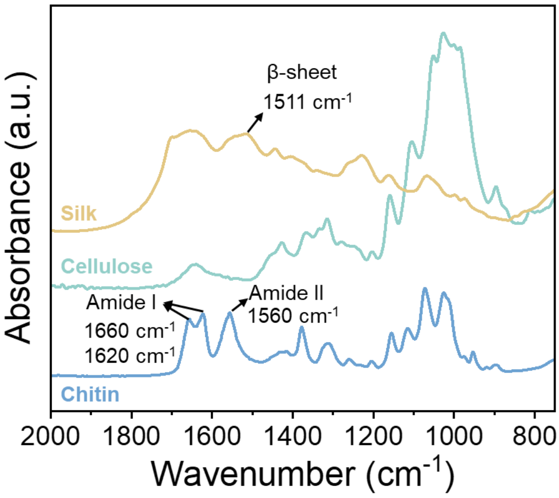

Figure S2. Fourier transform infrared (FT-IR)/attenuated total reflection spectra of chitin, cellulose, and silk nanofiber aerogels.

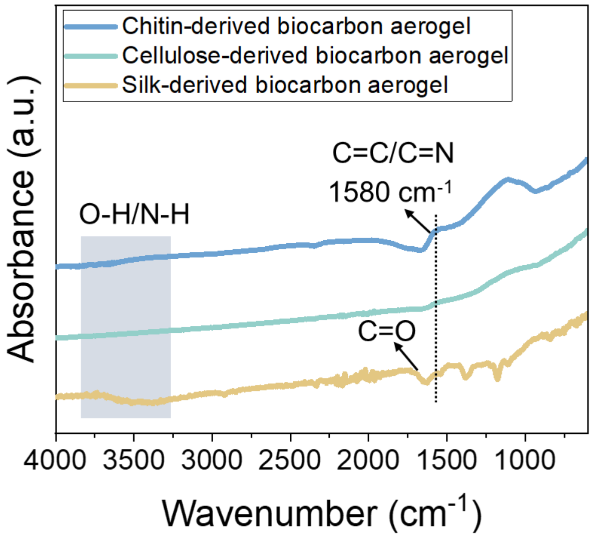

Figure S3. FT-IR/attenuated total reflection spectra of chitin, cellulose, and silk nanofiber–derived biocarbon aerogels.

The FT-IR spectra of the biocarbon aerogels showed weak O−H and N−H peaks, suggesting that pyrolysis resulted in the removal of H, N, and O and weakened hydrogen bonding between the nanofibers.[1] The peak around 1580 cm^{-1}, ascribed to C=C and/or C=N vibrations, indicated the progress of carbonization upon high-temperature pyrolysis.[2,3]

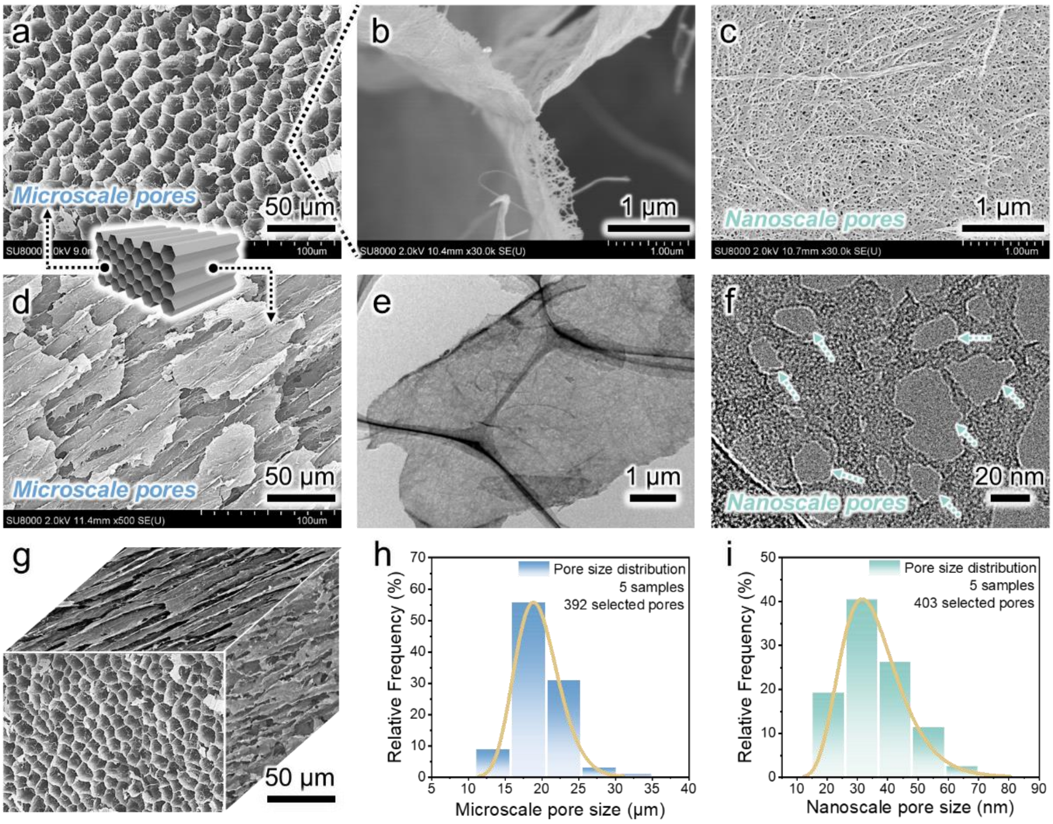

Figure S4. Field-emission scanning electron microscopy (FE-SEM) images of microscale pores in directions (a, b) parallel and (d) perpendicular to the anisotropic ordered pore structures and (c) nanoscale pores in the pore walls of the chitin nanofiber–derived biocarbon aerogel. (e, f) High-resolution transmission electron microscopy images of the nanoscale pores in the aerogel pore walls. (g) Schematic of the anisotropic ordered pore structures. Size distributions of the (h) microscale ordered pores and (i) nanoscale pores in the pore walls.

Field-emission scanning electron microscopy (FE-SEM) analysis revealed that the chitin nanofiber–derived biocarbon aerogel featured honeycomb-like pore structures (**Figure S4**a) formed by thin pore walls (Figure S4b) composed of interconnected nanofiber networks, with abundant in-plane nanopores observed between the nanofibers (Figure S4c). In addition, highly ordered channels were observed perpendicular to the direction of ice crystal growth (Figure S4d), which confirmed the formation of ordered porous channels after freeze-drying and pyrolysis. The presence of microscale pores in the main pore wall structures implied that the ordered pore walls were not completely continuous. The pore walls were further analyzed by low-magnification transmission electron microscopy (TEM), which confirmed that the pore wall was thin (Figure S4e) and featured nanoscale pores with sizes of 18–69 nm (Figure S4f).

Figure S4g presents a schematic of the microscale three-dimensional structures of the

biocarbon aerogel based on the results of FE-SEM and high-resolution TEM observations in different directions. The biocarbon aerogel had honeycomb-like anisotropic ordered pores with an average pore size of 19.5 ± 3.1 μm (Figure S4h) in one direction. In the other directions, ordered pore walls composed of nanofiber networks with nanopores of 35.6 ± 10.2 nm were observed (Figure S4i).

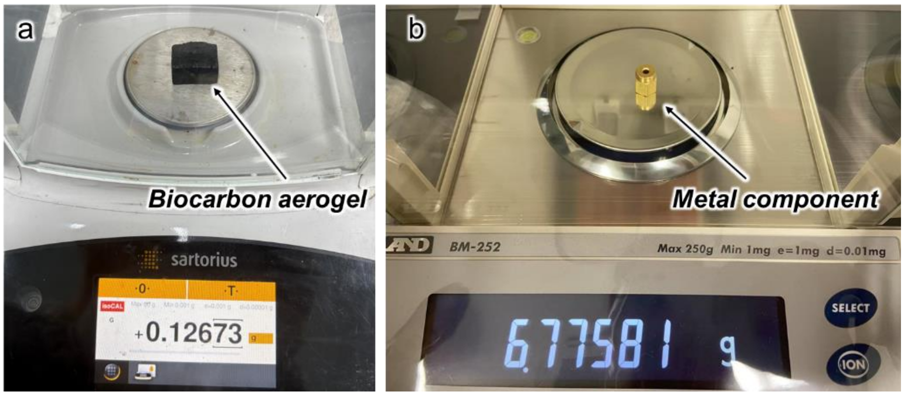

Figure S5. (a) Weight of the chitin nanofiber–derived biocarbon aerogel (0.13 g) and (b) metal object (6.8 g) used to demonstrate the elasticity of the former.

Table S1. Recovery speeds of the elastic chitin nanofiber–derived biocarbon aerogel and state-of-the-art elastic carbon aerogels.

Aerogel composition	Pore structure	Recovery speed [mm s^{-1}]	Ref.
Graphene-coated carbon nanotubes	Random	11.2	4
Graphene	Cell-like	117	5
Chitin derived carbon–coated graphene	Lamellar	590	6
GO/aminosilanol-cellulose nanofiber	Centripetal-like	792	7
Carbon nanotubes	Cross-linked	845	8
Graphene	foam-like	1084.6	9
Carbon nanotubes	Polar bear hair-like	1434	10
Chitin nanofiber–derived carbon	Honeycomb-like	1624	This work

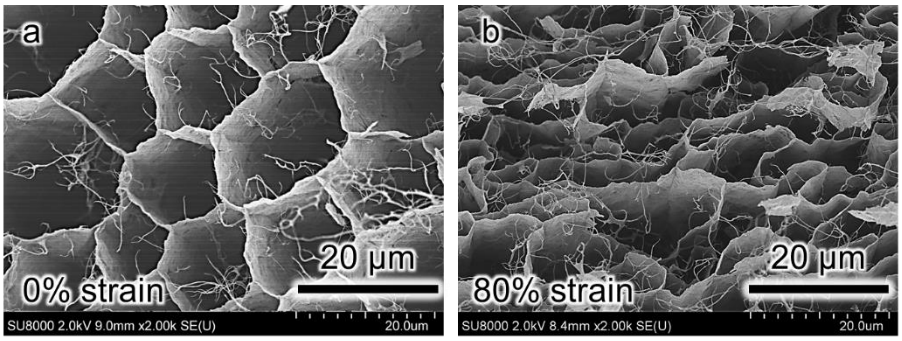

Figure S6. FE-SEM images of the chitin nanofiber–derived biocarbon aerogel in the direction parallel to the ordered pores at compression strains of (a) 0% and (b) 80%.

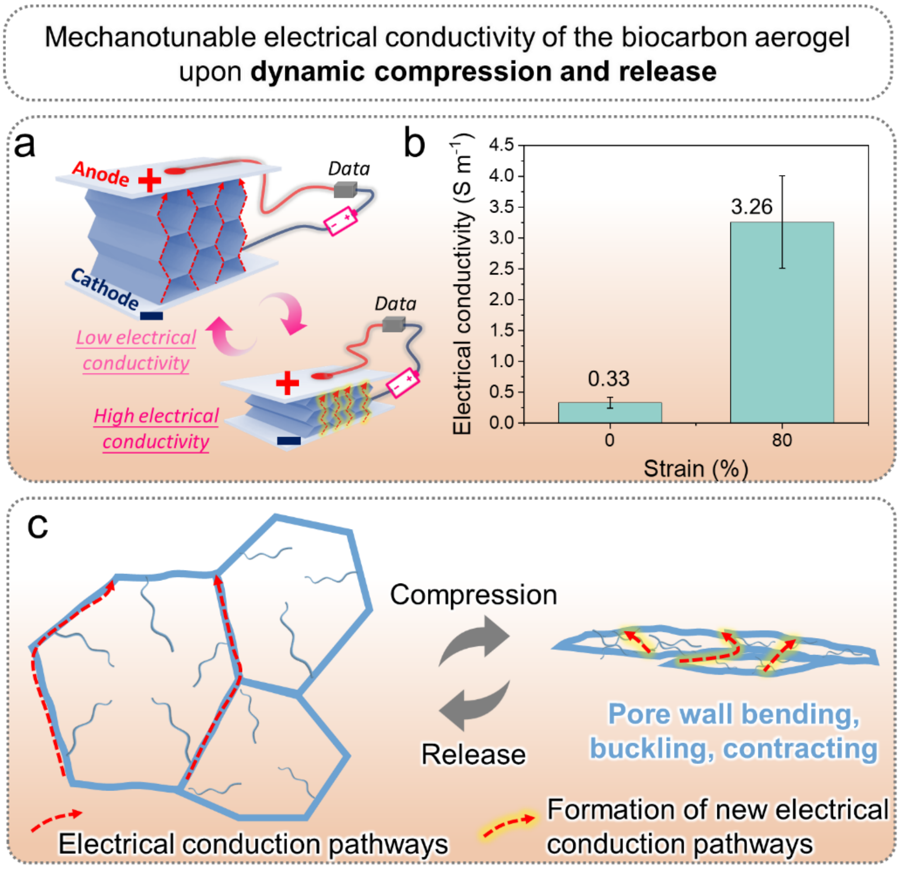

Figure S7. (a) Schematics and (b) electrical conductivities of the chitin nanofiber–derived biocarbon aerogel in compressed (80% strain) and uncompressed (0% strain) states. (c) Putative mechanism of the mechanotunable electrical conduction of the abovementioned aerogel.

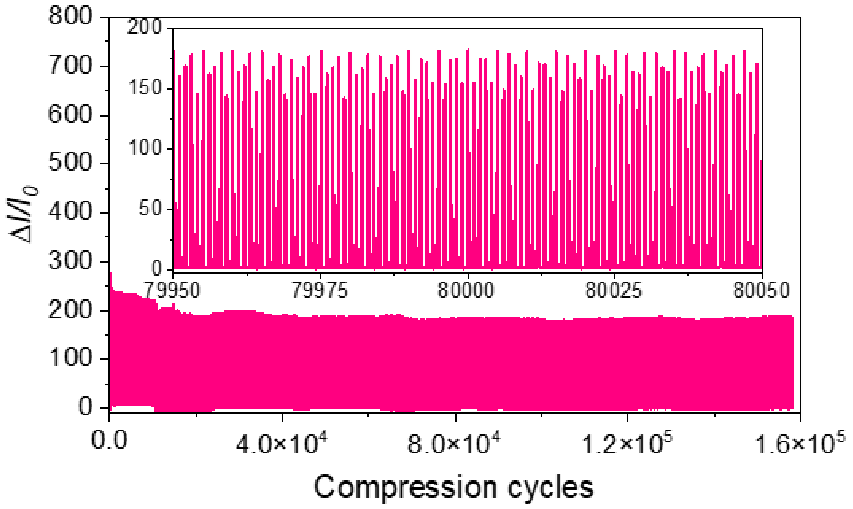

Figure S8. Relative current change ($\Delta I/I_0$) of the chitin nanofiber-derived biocarbon aerogel during 160,000 cycles of compression–release at 25 °C and 80% strain.

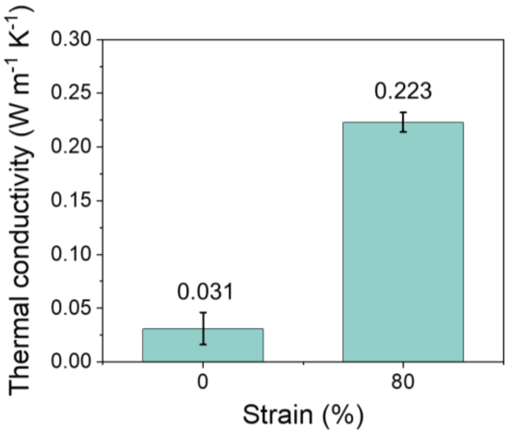

Figure S9. Through-plane thermal conductivities of the chitin nanofiber–derived biocarbon aerogel at compression strains of 0% and 80%.

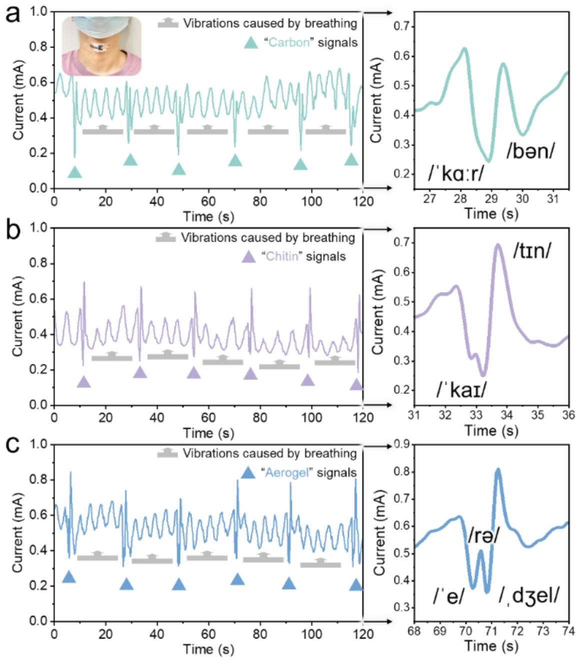

Figure S10. Real-time current changes recorded by the biocarbon-based pressure sensor upon the periodical pronunciation of (a) "carbon", (b) "chitin", and (c) "aerogel".

The compression of the chitin nanofiber–derived biocarbon aerogel induced by vocal cord vibrations enabled the detection of two characteristic current peaks corresponding to the two syllables of "chitin" and "carbon" (**Figure S10**a–b) and three peaks corresponding to the three syllables of "aerogel." These results demonstrate the applicability of the biocarbon aerogel–based pressure sensor for voice recognition devices.

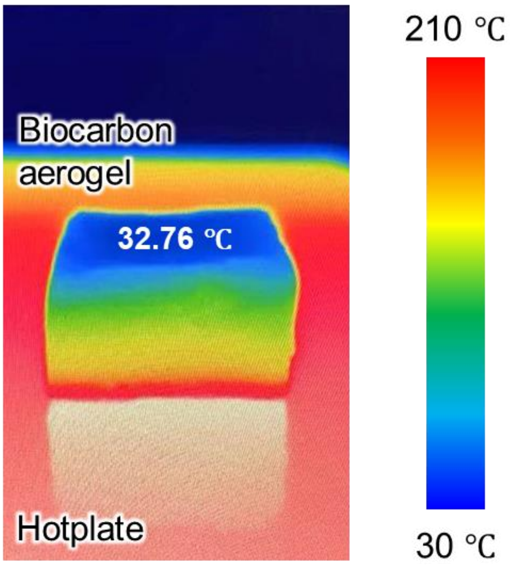

Figure S11. IR false-color image of the chitin nanofiber–derived biocarbon aerogel without a glass plate after unilateral heating at 200 °C for 5 min. Saturated top-surface temperature ≈ 33 °C.

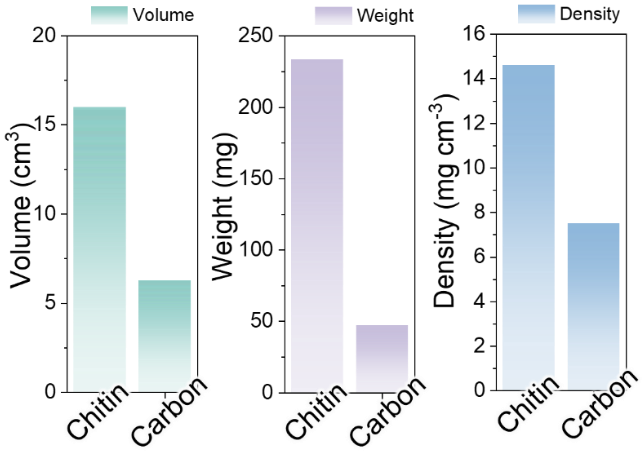

Figure S12. Volumes, weights, and densities of the chitin nanofiber–based aerogel and corresponding biocarbon aerogel.

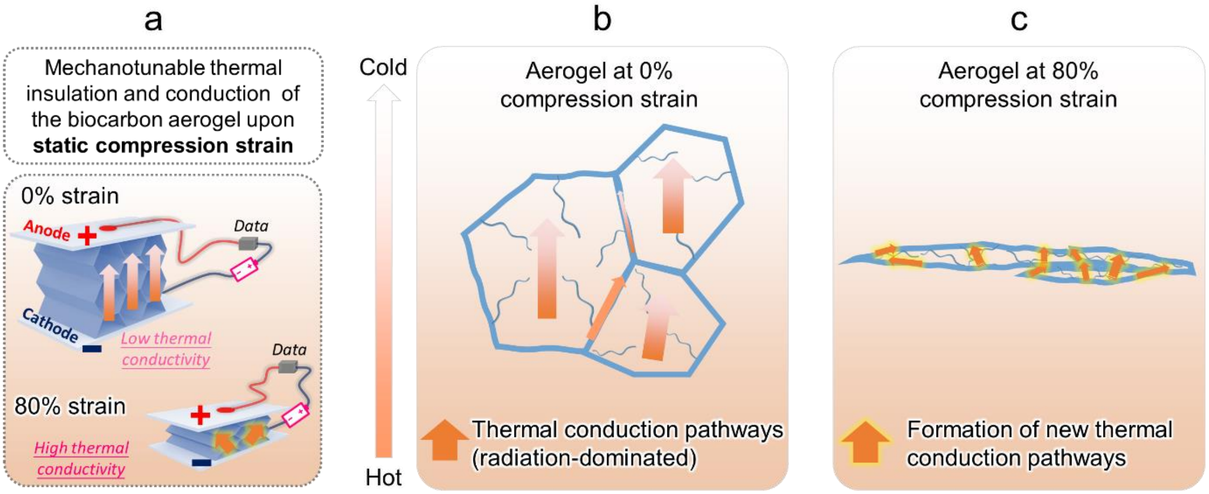

Figure S13. (a) Schematic behavior of the chitin nanofiber–derived biocarbon aerogel upon compression (80% strain) and release (0% strain). (b-c) Putative mechanism of the mechanotunable thermal conduction of the biocarbon aerogel upon compression and release.

References

(1) Hou, Y. Z.; Guan, Q. F.; Xia, J.; Ling, Z. C.; He, Z. Z.; Han, Z. M.; Yang, H. Bin; Gu, P.; Zhu, Y. B.; Yu, S. H.; Wu, H. A. Strengthening and Toughening Hierarchical Nanocellulose via Humidity-Mediated Interface. *ACS Nano* **2021**, *15* (1), 1310–1320. https://doi.org/10.1021/acsnano.0c08574.

(2) Nguyen-Thai, N. U.; Hong, S. C. Structural Evolution of Poly(Acrylonitrile-Co-Itaconic Acid) during Thermal Oxidative Stabilization for Carbon Materials. *Macromolecules* **2013**, *46* (15), 5882–5889. https://doi.org/10.1021/ma401003g.

(3) Li, X.; Zhu, L.; Kasuga, T.; Nogi, M.; Koga, H. All-Nanochitin-Derived, Super-Compressible, Elastic, and Robust Carbon Honeycombs and Their Pressure-Sensing Properties over an Ultrawide Temperature Range. *ACS Appl. Mater. Interfaces* **2023**, *15* (35), 41732–41742.

(4) Kim, K. H.; Oh, Y.; Islam, M. F. Graphene Coating Makes Carbon Nanotube Aerogels Superelastic and Resistant to Fatigue. *Nat. Nanotechnol.* **2012**, *7* (9), 562–566. https://doi.org/10.1038/nnano.2012.118.

(5) Qiu, L.; Liu, J. Z.; Chang, S. L. Y.; Wu, Y.; Li, D. Biomimetic Superelastic Graphene-

Based Cellular Monoliths. *Nat. Commun.* **2012**, *3*, 1241. https://doi.org/10.1038/ncomms2251.

(6) Gao, H. L.; Zhu, Y. B.; Mao, L. B.; Wang, F. C.; Luo, X. S.; Liu, Y. Y.; Lu, Y.; Pan, Z.; Ge, J.; Shen, W.; Zheng, Y. R.; Xu, L.; Wang, L. J.; Xu, W. H.; Wu, H. A.; Yu, S. H. Super-Elastic and Fatigue Resistant Carbon Material with Lamellar Multi-Arch Microstructure. *Nat. Commun.* **2016**, *7*, 12920. https://doi.org/10.1038/ncomms12920.

(7) Wu, X.; Liu, T.; Qiu, Y.; Hou, Z.; Cai, C.; Li, M.; Xu, Y.; Mou, Y.; Luo, S.; Lu, D. Elastic Yet Strength Triboelectric Aerogel Enabled by Constructing a Supramolecular System. *Adv. Funct. Mater.* **2025**, *35* (11), 2417067. https://doi.org/10.1002/adfm.202417067.

(8) Zhuang, L.; Lu, D.; Zhang, J.; Guo, P.; Su, L.; Qin, Y.; Zhang, P.; Xu, L.; Niu, M.; Peng, K.; Wang, H. Highly Cross-Linked Carbon Tube Aerogels with Enhanced Elasticity and Fatigue Resistance. *Nat. Commun.* **2023**, *14* (1), 3178. https://doi.org/10.1038/s41467-023-38664-6.

(9) Lv, L.; Zhang, P.; Xu, T.; Qu, L. Ultrasensitive Pressure Sensor Based on an Ultralight Sparkling Graphene Block. *ACS Appl. Mater. Interfaces* **2017**, *9*, 22885–22892. https://doi.org/10.1021/acsami.7b07153.

(10) Zhan, H.; Wu, K.; Zhan, H.; Wu, K.; Hu, Y.; Liu, J.; Li, H.; Guo, X.; Xu, J.; Yang, Y. Biomimetic Carbon Tube Aerogel Enables Super-Elasticity and Thermal Insulation. *Chem* **2019**, *5* (7), 1871–1882. https://doi.org/10.1016/j.chempr.2019.04.025.
